# CDKL5 and Shootin1 Interact and Concur in Regulating Neuronal Polarization

**DOI:** 10.1371/journal.pone.0148634

**Published:** 2016-02-05

**Authors:** Mohammad Sarfaraz Nawaz, Elisa Giarda, Francesco Bedogni, Paolo La Montanara, Sara Ricciardi, Dalila Ciceri, Tiziana Alberio, Nicoletta Landsberger, Laura Rusconi, Charlotte Kilstrup-Nielsen

**Affiliations:** 1 Laboratory of Genetic and Epigenetic Control of Gene Expression, Department of Biotechnology and Life Sciences, Centre of Neuroscience, University of Insubria, 21052 Busto Arsizio, Italy; 2 San Raffaele Rett Research Unit, Division of Neuroscience, San Raffaele Scientific Insitute, 20132 Milan, Italy; 3 Molecular Histology and Cell Growth Unit, Fondazione Istituto Nazionale Genetica Molecolare (INGM), 20122 Milan, Italy; 4 Laboratory of Biochemistry and Functional Proteomics, Department of Science and High Technology, Centre of Neuroscience, University of Insubria, 21052 Busto Arsizio, Italy; 5 Department of Medical Biotechnology and Translational Medicin, University of Milan, 20090 Segrate, Italy; University of Wurzburg, GERMANY

## Abstract

In the last years, the X-linked cyclin-dependent kinase-like 5 (*CDKL5*) gene has been associated with epileptic encephalopathies characterized by the early onset of intractable epilepsy, severe developmental delay, autistic features, and often the development of Rett syndrome-like features. Still, the role of CDKL5 in neuronal functions is not fully understood. By way of a yeast two hybrid screening we identified the interaction of CDKL5 with shootin1, a brain specific protein acting as a determinant of axon formation during neuronal polarization. We found evidence that CDKL5 is involved, at least in part, in regulating neuronal polarization through its interaction with shootin1. Indeed, the two proteins interact in vivo and both are localized in the distal tip of outgrowing axons. By using primary hippocampal neurons as model system we find that adequate CDKL5 levels are required for axon specification. In fact, a significant number of neurons overexpressing CDKL5 is characterized by supernumerary axons, while the silencing of CDKL5 disrupts neuronal polarization. Interestingly, shootin1 phosphorylation is reduced in neurons silenced for CDKL5 suggesting that the kinase affects, directly or indirectly, the post-translational modification of shootin1. Finally, we find that the capacity of CDKL5 to generate surplus axons is attenuated in neurons with reduced shootin1 levels, in agreement with the notion that two proteins act in a common pathway. Altogether, these results point to a role of CDKL5 in the early steps of neuronal differentiation that can be explained, at least in part, by its association with shootin1.

## Introduction

During the last years, the cyclin-dependent kinase-like 5 gene (*CDKL5*; OMIM 300203), located on the X-chromosome, has been associated with severe encephalopathy associated with X-linked infantile spasms and some Rett syndrome (RTT)-like features [1,2]. CDKL5 is a serine/threonine kinase with an N-terminal catalytic domain that shares homology with the mitogen-activated protein kinases and cyclin-dependent kinases [3]. The long C-terminal region of CDKL5 regulates several aspects of its properties such as catalytic activity, subcellular localization, and protein stability [4–6].

In accordance with its involvement in intellectual disability, CDKL5 is highly abundant in brain where it is significantly induced in early post-natal stages suggesting a role in brain maturation [7,8]. The high levels of CDKL5 in brain can be attributed predominantly to neuronal expression since only low levels are present in glial cells. CDKL5 is involved in neuronal morphogenesis and dendritic arborization in a Rac1 dependent manner, as shown by its silencing in primary neurons [9]. Moreover, proper spine development and synapse formation was found to depend on the presence of functional CDKL5 in accordance with its accumulation in the post-synaptic densities of mature neurons [10,11]. Recently, two knockout mouse models of *Cdkl5* have been developed [12,13]. These mice do not show gross general changes or brain morphology defects, but exhibit reduced dendritic branching and outgrowth, together with defects in neural circuit communication. Interestingly, both *Cdkl5*-null mouse models display some behavioral deficits such as autistic-like features and impaired learning and memory while spontaneous seizures, which represent the most striking phenotype in humans, are absent. Despite a clear involvement of CDKL5 in shaping the dendritic arbor and synaptic spines, its role in the early phases of neuronal development, when the axon is specified, is poorly described.

Primary hippocampal neurons have been instrumental to identify cellular and molecular changes involved in generating neuronal polarity [14,15], leading to the establishment of one axon and several dendrites per neuron. Dissociated embryonic hippocampal neurons form several immature neurites upon plating. The early steps of their spontaneous polarization involve the breaking of neuronal symmetry leading to the accumulation of distinct proteins in the axon-to-be [16]. Here we present evidence that both increased and reduced levels of CDKL5 are detrimental for the correct progression of these events, clearly suggesting a role of the kinase in the proper establishment of neuronal polarity. This effect depends, at least in part, on the association of CDKL5 with shootin1, a key protein in neuronal polarization [17,18]. Accordingly, neurons with altered CDKL5 levels show polarization defects that partially overlap those of shootin1. Altogether, our data provide novel insights in the role of CDKL5 for proper brain functions and suggest that the loss of CDKL5 might impact neuronal differentiation during very early developmental stages.

## Materials and Methods

### Ethics Statement

Protocols and use of animals were approved by the Animal Ethics Committee of the University of Insubria and in accordance with the guidelines released by the Italian Ministry of Health. Adult mice were euthanized by cervical dislocation, while neonates were sacrificed by exposure to CO_2_ followed by decapitation.

### Reagent and Antibodies

The following primary antibodies were used in immunofluorescence and western blotting experiments: custom-made immunopurified anti-CDKL5, used for coimmunoprecipitation experiments, has been described elsewhere [8]. Anti-CDKL5 (HPA002847, Sigma-Aldrich), chicken anti-GFP (A10262, Life Technologies), anti-P-ERK (9101S, Cell Signaling), anti-shootin1 (PA5-17167 (rabbit) Thermo; 3279 (rabbit) Cell Signaling; sc244129 (goat) Santa Cruz), anti-Tau1 (MAB3420, Millipore), anti-MAP2 (ab11267, Abcam), anti-NFL (MA5-14981, Thermo Scientific), anti-Tuj1 (MMS-435P, Covance), anti-β-actin (A5441, Sigma-Aldrich). DAPI and secondary Alexa Fluor anti-rabbit, anti-mouse, anti-chicken and anti-goat antibodies for immunofluorescence experiments were purchased from Life Technologies. HRP-conjugated secondary anti-mouse, anti-rabbit, and anti-goat antibodies for western blottings were obtained from Thermo Scientific.

### Yeast Two-Hybrid Screening

The yeast two-hybrid screening was performed by Hybrigenics Services, S.A.S, Paris, France (http://www.hybrigenics-services.com). The C-terminus of human CDKL5 (amino acids 299–1030; GenBank accession number gi: 83367068) fused to the Gal4 DNA-binding domain and expressed from the inducible pB35 vector, was used as bait to screen a random-primed Human Adult Brain cDNA library constructed into pP6.

113 million clones (11-fold the complexity of the library) were screened using a mating approach with Y187 (MATα Gal4Δ Gal80Δ ade2-101 his3 leu2-3,-112 trp1-901 ura3-52 URA3::UASGAL1-LacZ, (met-)) and CG1945 (MATa Gal4-452 Gal80-538 ade2-101 his3-D200 leu2-3,112 trp1-901 ura3-52 lys2-801 URA3::Gal4 17mers (X3)-CyC1TATA-LacZ lys2::GAL1UAS-GAL1TATA-HIS3 cyhR) yeast strains. A total of 171 His^+^ colonies were selected on a medium lacking tryptophan, leucine, methionine and histidine. The prey fragments of the positive clones were amplified by PCR and sequenced at their 5’ and 3’ junctions. The resulting sequences were used to identify the corresponding interacting proteins in the GenBank database (NCBI) using a fully automated procedure. A confidence score (PBS, for Predicted Biological Score) was attributed to each interaction as previously described [19].

### Plasmids and Constructs

Flag-CDKL5 (107 kDa isoform) has been described elsewhere [6]. pCAGGS-CDKL5-ires-GFP was cloned by inserting an EcoRI-EcoRV digested PCR product containing the murine CDKL5 cDNA (NP_001019795) into pCAGGS-ires-GFP digested with EcoRI and SmaI. The K42R derivative was generated by site-directed mutagenesis using the QuickChange site-directed mutagenesis kit (Stratagene). pCAGGS-shootin1-ires-GFP was cloned likewise by inserting PCR amplified human shootin1 (NM_018330) into the EcoRI-SmaI digested vector. Lentiviral vectors to silence endogenous CDKL5 and shootin1 were generated by cloning double stranded oligonucleotides into the HpaI and XhoI sites of pLentiLox 3.7 (pLL 3.7). The target sequences of the shRNAs are as follows: shCDKL5#1: CTATGGAGTTGTACTTAA; shCDKL5#2: GTGAGAGCGAAAGGCCTT; shCDKL5#3: GCAGAGTCGGCACAGCTAT; shShootin1#1: 5’-CCACGGTGAATAAATAGAAAT (against the 3’UTR); shShootin1#2: GCAGAACCATCTTGAAATA; a shRNA against LacZ was used as control. All PCR derived constructs were sequence verified.

### Primary Neuronal Cultures

Primary hippocampal cultures were prepared from embryonic day 16 (E16) CD1 mouse embryos, considering the day of the vaginal plug as E0, as described previously [5] and plated on poly-L-lysine coated dishes at different densities: 2x10^3^/cm^2^ for immunostainings and 1x10^4^/cm^2^ for western blots. For overexpression studies, hippocampal neurons were transfected at 0 days in vitro (DIV0) by nucleofection using the Amaxa Basic Neuron Small Cell Number Nucleofector Kit following the manufacturer’s instructions. Embryos at E17 were used in this case to make neurons more resistant to the nucleofection process. Infection of hippocampal and cortical neurons was performed by adding lentiviral particles to the neurons before plating or 10 h after nucleofection.

### Immunoprecipitations

Immunoprecipitation of endogenous CDKL5 from P7 mouse brains was performed by homogenizing the whole brain in lysis buffer (50 mM Tris-HCl pH 7.5; 150 mM NaCl, 1 mM EDTA, 1mM EGTA, 1% Triton X-100, 0.1% SDS, 2 mM sodium pyrophosphate, 1 mM sodium orthovanadate), incubation on ice for 20 min and centrifugation at 100.000 g for 30 min at 4°C. The mouse brain extract (500 μg) was incubated overnight at 4°C with 20 μl purified anti-CDKL5 [8] or unrelated IgGs as control. The immunocomplexes were precipitated with protein-G Agarose (Life Technologies), washed with lysis buffer and analyzed by SDS-PAGE. Shootin1 was immunoprecipiated from P5 brains lysed in the above lysis buffer (w/o SDS) with anti-shootin1 (Thermo Scientific) or unrelated IgGs as control. The protein-G agarose resin was saturated with 5% BSA in PBS before being added to the immunocomplexes. Goat anti-shootin1 was used to detect immunoprecipitated shootin1.

Exogenously expressed Flag-CDKL5 was immunoprecipitated from transfected HeLa cells lysed in lysis buffer (NaCl 150 mM, Trish pH 8 50 mM, Triton 1%, EDTA 2 mM with protease inhibitor cocktail (Sigma Aldrich) and PhosStop (Roche)). The sonicated lysate was centrifugated and the obtained whole cell extract pre-cleared with Protein-G agarose (Thermo Scientific). The anti-Flag resin (Sigma Aldrich) was blocked with 5% BSA in PBS for 4 h where after it was added to the pre-cleared extract. After over night incubation the immunocomplexes were washed several times with wash buffer (400 mM NaCl, 50 mM Tris-HCL pH 8, 1% Triton, 2 mM EDTA, protease inhibitor cocktail), once with PBS and separated on a 8% SDS-PAGE.

### Immunofluorescence

Primary neurons were fixed in 4% paraformaldehyde for 10 min, blocked for 1 h (5% horse serum, 0.2% Triton X-100, phosphate buffer), then incubated overnight at 4°C with the primary antibody in phosphate buffer, 5% horse serum and 0.2% Triton X-100 and finally with the corresponding secondary antibodies. Nuclei were stained with DAPI and the cells mounted for microscopy analysis at a Nikon Eclipse Ni microscope equipped with a Nikon Digital Sight DS-2MBWc camera. Neuronal polarization was analyzed by staining with Tau1 and MAP2 (axon and dendrite specific markers, respectively). Neurites longer than 100 μm in which the intensity of Tau-1 staining increased significantly along the proximal to distal axis were counted as axons. According to this parameter, neurons were classified either as lacking an axon (no axon) when none of the neurites could be considered an axon or with multiple axons when more than one neurite showed the properties of an axon. Image analysis was performed with Adobe Photoshop and IMAGE J (http://rsbweb.nih.gov/ij/).

### In situ hybridization

Antisense riboprobes against mouse *Cdkl5* and *shootin1* (both full length) were used to stain for endogenous mRNAs. In situ hybridizations were run on slide mounted cryostat sections (12 μm) using a protocol based on a previous published paper [20].

### Two-Dimensional Isoelectric Focusing

2.5x10^6^ cortical neurons isolated from E16 mouse embryos were infected at DIV0 to silence CDKL5 expression. At DIV7, cells were washed and lysed in UTC buffer (7 M urea, 2 M thiourea, 4% CHAPS). Approximately 200 μg of extract were loaded on 7 cm IPG DryStrips with a linear 4–7 pH gradient and isoelectric focusing performed with an IPGphor II apparatus (GE Healthcare) according to the manufacturer’s instructions. Subsequent SDS-PAGE was performed using 8% gels and proteins were detected by western blotting. Phosphatase treatment was done by adding 100 units of Lambda Protein Phosphatase (Lambda PP; New England Biolabs) to the extract diluted in 2 ml 0.1% SDS, 0.1 mM MnCl_2_, and 1x phosphatase-buffer. After an overnight incubation at 30°C the samples were concentrated with Ultracel 10K centrifugal filters (Millipore) and subjected to isoelectric focusing.

### Virus Preparation

HEK293T cells, grown in 150 mm dishes, were cotransfected with the packaging vectors pVSV-G, pMDL, pREV and either pLL3.7-shCDKL5#1, pLL3.7-shCDKL5#2, pLL3.7-shCDKL5#3, pLL3.7-shShootin#1, pLL3.7-shShootin#2, or pLL3.7-shLacZ using calcium phosphate. The viral particles were harvested 36 h post-transfection and concentrated by ultracentrifugation with SW32 rotor (Beckman Instruments) at 20.000 rpm for 2 h. Viruses were resuspended in PBS and stored at -80°C.

### Statistical Analysis

Values are expressed as means ± standard error (SEM). The significance of results was obtained by Student’s *t* test and ANOVA two-way followed by Tuckey’s post-hoc test. Statistical significance was established as *p* < 0.05.

## Results

### Shootin1 is a novel interactor of CDKL5 in vivo

**Fig 1:**
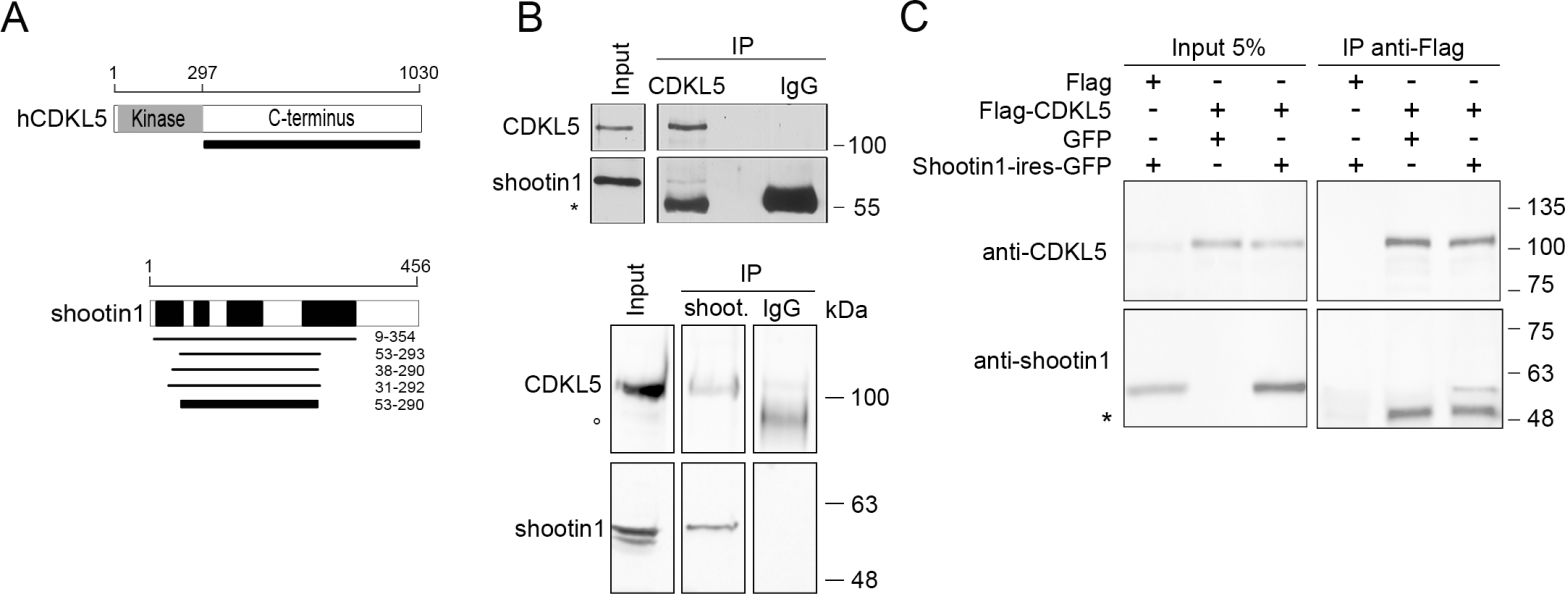
CDKL5 interacts with shootin1 in vivo. (A) A yeast two-hybrid screening identified shootin1 as a CDKL5 interacting protein. The C-terminal region of hCDKL5, spanning amino acids 299–1030, was used as bait (upper, thick bar). The diagram below shows shootin1 with its coiled coil domains in black. The clones identified in the screen are indicated as black bars and the minimum CDKL5 interacting region as a black bar. (B) Coimmunoprecipitation of P5-7 brain lysates with anti-CDKL5 (upper, n = 3) or anti-shootin1 (lower, n = 3) antibodies (both rabbit). IgGs were used as negative control. The immunoprecipitates and inputs (5% of the brain lysates) were analyzed by immunoblotting for CDKL5 and shootin1 (using a goat anti-shootin1 antibody). Asterisks indicate the immunoglobulin heavy chains and the open circle an unspecific band detected with anti-CDKL5. (C) Coimmunoprecipitation of HeLa cells overexpressing either Flag-CDKL5 or shootin1 or both proteins together. Whole cell lysates were immunoprecipiated with an anti-Flag resin and inputs (5%) and immunocomplexes analyzed by western blotting as indicated. Asterisk shows an anti-shootin1 reactive protein that copurifies with CDKL5. (n = 3).

To elucidate the neuronal functions of CDKL5 we searched for interacting proteins through a yeast two-hybrid screening. The C-terminal region of human CDKL5 (amino acids 299–1030) regulates its activities and serves as interfase with MeCP2, DNMT1, and PSD95 [7,21,11]. We therefore used this region as bait to screen a human adult brain cDNA library. We obtained 171 interacting clones of which shootin1 obtained the highest confidence score. Shootin1 was previously described as a brain-specific protein that is involved in axon formation and neuronal polarization [17,18]. A total of seven clones containing different but overlapping regions of shootin1 were picked in the screening enabling us to map the central region, spanning amino acids 53–290, as the surface that contacts CDKL5 (Fig 1A). To confirm a physical interaction between the two proteins in vivo, lysates from P7 brains were used to immunoprecipitate CDKL5. Subsequent western blotting specifically detected shootin1 in the CDKL5 immunoprecipiate (Fig 1B, upper panel). The reciprocal experiment, in which CDKL5 copurified with shootin1 (Fig 1B, lower panel), confirmed that the two proteins associate in a common complex in mouse brain. Further evidence of the capacity of CDKL5 and shootin1 to interact was obtained by coimmunoprecipitating the overexpressed proteins from transfected HeLa cells (Fig 1C).

**Fig 2:**
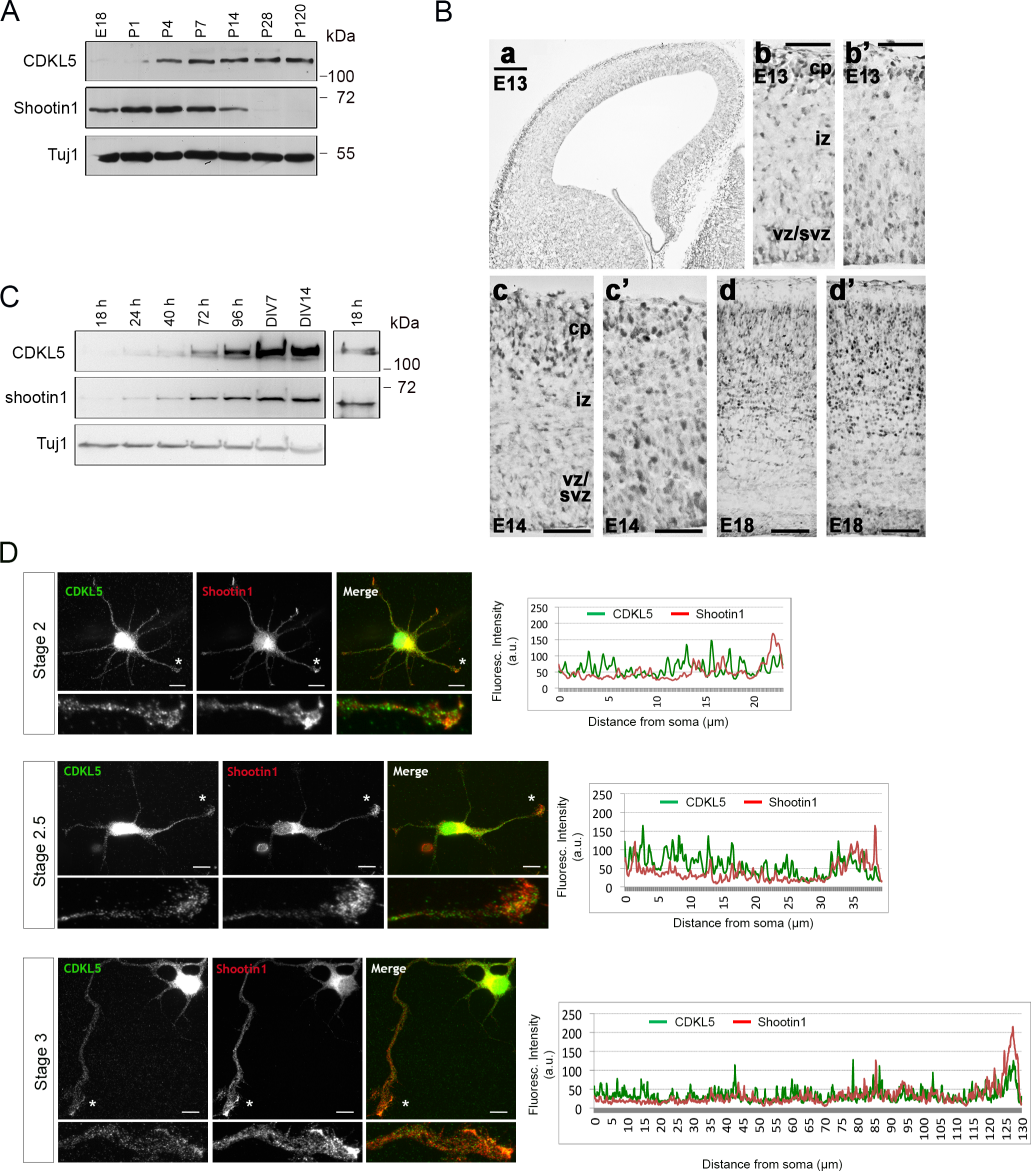
CDKL5 and shootin1 are coexpressed in brains and neurons. (A) Western blot analysis showing CDKL5 and shootin1 levels in mouse brain at the indicated developmental stages using Tuj1 as loading control. (n = 2) (B) *Shootin1* is expressed in the cortex, as early as E13, in the cortical plate (cp) and its levels increase ongoing with development (a,b,c,d); *Cdkl5* (b’,c’,d’) follow the same pattern. Low but detectable levels of *shootin1* and *Cdkl5* mRNAs are present in cells migrating out of the ventricular and sub-ventricular zone (vz-svz) towards their final destination in the cortical plate (b,b’). At E18 *shootin1* and *Cdkl5* are strongly expressed throughout the whole thickness of the cortex (d,d’). Scale bars: 50 μm: b,b’,c,c’; 100 μm: d,d’; 200 μm: a. (C) Western blot showing CDKL5 and shootin1 levels in cultured primary hippocampal neurons at the indicated stages. A longer exposure of the 18 h time point is shown to the right. (n = 2). (D) Immunofluorescence analysis (left) of hippocampal neurons at stages 2–3 with antibodies against CDKL5 (green) and shootin1 (red). The small panels show the magnification of the minor processes/axons indicated with asterisks. Quantitative profiles showing the fluorescence intensities of shootin1 (red) and CDKL5 (green) from the soma to the distal tip of the neurites/axons indicated with asterisks are shown to the right. Scale bar: 10 μm.

To evaluate when a physical association between the two proteins might exist in vivo, we compared their temporal expression pattern during brain maturation. By immunoblotting lysates of mouse brains at different developmental stages we observed that shootin1 was strongly expressed from E18 until P7 when its levels started declining becoming undetectable in the adult brain (Fig 2A). In accordance with our previous results, CDKL5 expression was strongly induced in the first post-natal days [5] and a prominent coexpression of the two proteins was observed in the early post-natal stages from P4 to P14.

We then compared the expression pattern of *Cdkl5* and *shootin1* in the developing cortex by in situ hybridization (Fig 2B). In line with a previous report [22], *shootin1* transcripts were detected as early as E13 where its expression overlapped with that of *Cdkl5* in post-mitotic cortical neurons both migrating out of the proliferative zone (ventricular and sub-ventricular zones, VZ and SVZ) and residing in the cortical plate (CP). At E18 high levels of both *shootin1* and *Cdkl5* mRNAs were detected in the cortex, further suggesting that shootin1 and CDKL5 may be expressed by the same neurons, supporting the notion of their in vivo interaction.

To assess in more details whether both CDKL5 and shootin1 are physically present in neurons during the first stages of neuronal differentiation, we analyzed their expression in primary hippocampal neurons prepared from E16 mouse brains and collected at different time points (Fig 2C). Both shootin1 and CDKL5 levels are low but detectable 18 h after plating but are concomitantly induced between 40–72 h of culturing and reach a plateau of expression at DIV7.

We next analyzed the localization of the two proteins in cultured hippocampal neurons at the initial stages after plating (Fig 2D). In stage 2 neurons, before axon specification occurs, we detected shootin1 in the distal tip of minor processes, as expected, besides its presence in the nucleus and soma [17]. Later, when the axon has been specified (stage 2.5) and when axon outgrowth is clearly detectable (stage 3), shootin1 presented a clear accumulation in the axonal growth cone. Although CDKL5 did not accumulate in the distal tip of minor processes and axonal growth cones, it could easily be detected all along these processes from the soma to the distal tip (S1 Fig) where it partially colocalized with shootin1 (Fig 2D).

Altogether, these data indicate the existence of a concurrent spatial and temporal expression profile for CDKL5 and shootin1, supporting the notion of a physical interaction between the two proteins.

### Neuronal polarization depends on proper CDKL5 levels

**Fig 3:**
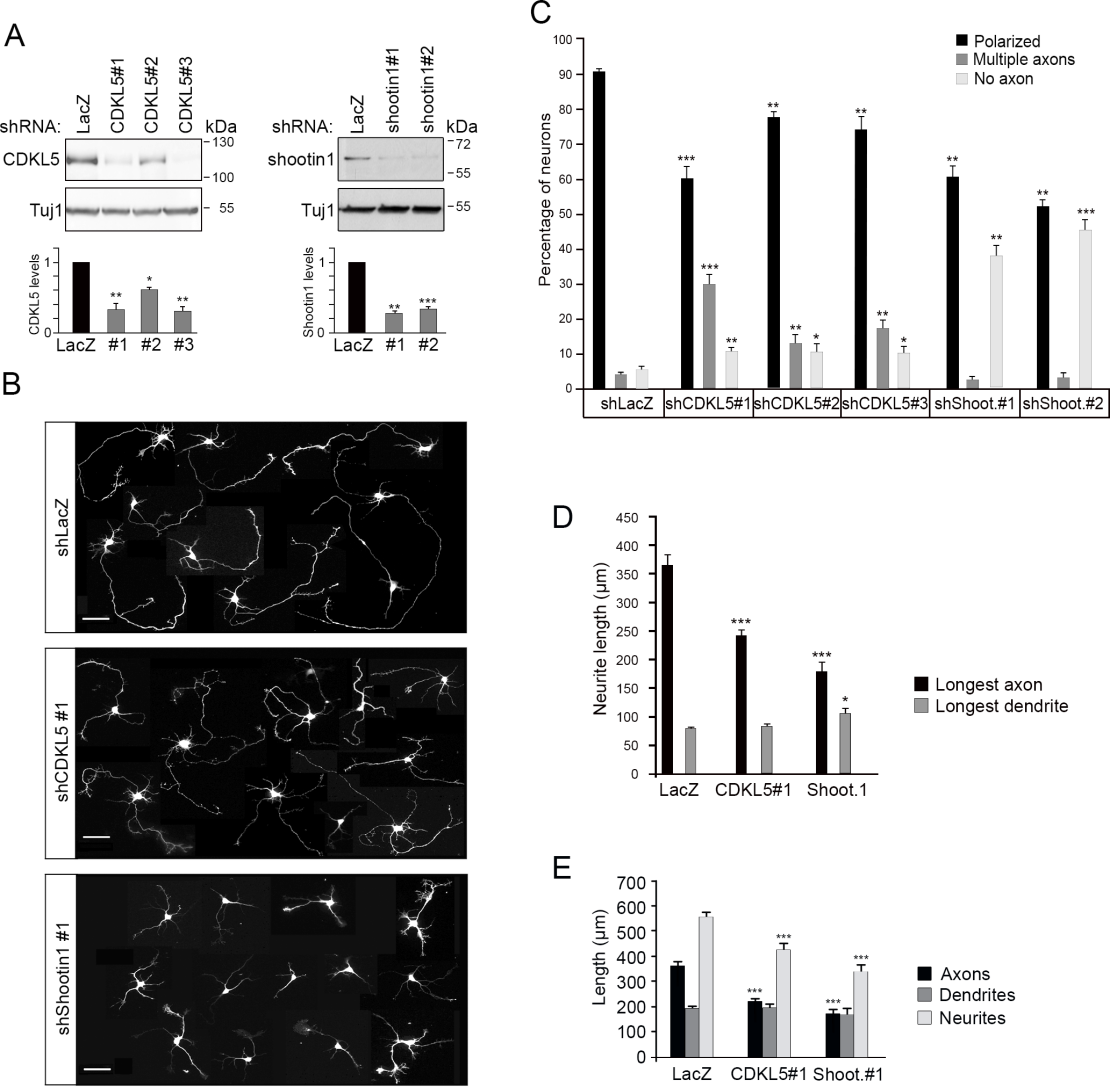
CDKL5 knock-down causes aberrant neuronal polarization. (A) Western blot showing CDKL5 and shootin1 levels in primary hippocampal neurons infected with shRNAs against CDKL5, shootin1 or, as control, LacZ. Neurons were infected at DIV0 and cell lysates prepared after 96 h. Tuj1 was used as loading control. The graph shows the quantified protein levels as means of n≥3 experiments, *p<0,05, **p<0,01, ***p<0,001 (Student’s *t* test). (B) Representative images of neurons 96 h after plating and infection of viral particles expressing GFP together with shRNAs against LacZ, CDKL5 or shootin1. The image is a composed panel of GFP-positive neurons. Scale bar: 50 μm. (C) Graph showing polarization of neurons expressing shRNAs against LacZ, CDKL5 or shootin1. Polarized neurons are indicated with black bars, those with numerous axons with dark grey bars and neurons with no axon with light grey bars. Data present percentage of neurons as means ±SEM (n≥250 neurons/condition from n≥3 experiments), *p<0,05, **p<0,01, ***p<0,001 (Student’s *t* test). (D) Quantification of the length of the longest axon and dendrite of shRNA-expressing neurons. Data present neurite length as means ±SEM (a total of 110 neurons were analyzed in 4 independent experiments). (E) Neurite length of GFP-positive neurons bearing one axon (a total of 74 neurons were analyzed in 3 independent experiments). Data in D and E present neurite length as means ±SEM, *, p<0,05, ***p<0.001. (Student’s *t* test).

Shootin1 has a well-established role in regulating axon formation. Its interaction with CDKL5 prompted us to study the role of the kinase for neuronal polarization using loss- and gain-of-function approaches in hippocampal neuronal cultures. We first analyzed the effect of reduced CDKL5 levels for neuronal polarization. CDKL5, shootin1 or, as control, LacZ were silenced by infecting primary hippocampal cultures, prepared from E16 mouse embryos, with lentiviral particles expressing specific shRNAs. The efficient silencing of CDKL5 and shootin1 was verified by immunoblotting lysates prepared 96 h after infection with three shRNAs against CDKL5 and two against shootin1 (Fig 3A). At DIV4, infected GFP-positive neurons silenced for CDKL5 (Fig 3B) presented an altered morphology as compared to controls. As expected, neurons silenced for shootin1 appeared severely stunted, lacking any long process and displaying several short neurites.

We then analyzed how the loss of CDKL5 or shootin1 influenced axon specification by immunostaining with the axonal and dendritic markers, Tau1 and MAP2, respectively. Correctly polarized neurons were recognized as bearing a single axon with a strong distal staining for Tau1 and several minor MAP2 positive neurites, whereas others contained multiple axons or no axon (S2 Fig). As expected, at DIV4, axon specification had occurred in the vast majority of control neurons (means ±SEM: 90.32±0.63%) that extended a single axon with an average length of 366±17.7 μm and several minor neurites (Fig 3C and 3D). In accordance with previous publications [17], the silencing of shootin1 interfered with axon specification and less than 60% of neurons carried one single, shorter axon (average length = 176±19.7 μm). Importantly, the silencing of CDKL5 with all three shRNAs also significantly interfered with neuronal polarization (polarized neurons means ±SEM: 59.53±3.42, 76.68±1.85 and 73.6±3.6% with shCDKL5 #1, #2 and #3, respectively). CDKL5 silencing increased the percentage of neurons bearing no axon (10.67±0.95, 10.35±2.25 and 10.08±1.61%, respectively, with shCDKL5 #1, #2 and #3). However, the depletion of CDKL5 also gave rise to neurons with supernumerary Tau1-positive and MAP2-negative axons (means ±SEM: 29.80±3.21, 12.90±1.95 and 16.58±2.42% with shCDKL5 #1, #2, #3, respectively) (Fig 3B and 3C; S2 Fig). The mean length of the longest axon in this group of CDKL5-depleted neurons was significantly shorter than that of control neurons (240±11.1 μm; Fig 3D). Since this might be caused by the presence of a surplus of axons limiting the amount of structural components, we also measured axon length in shCDKL5#1-expressing neurons bearing only one axon (Fig 3E). Also in this case, axon length was significantly reduced (shLacZ: 366±17.7 μm; shCDKL5#1: 223±11.2 μm) suggesting that CDKL5 is required for both axon specification and axonal outgrowth. The effect of reduced shootin1 levels on axon specification was only temporary since by DIV7 polarization levels similar to those of control cells was restored [17]. On the other hand, we found that the impairment caused by CDKL5 silencing persisted until DIV7 (polarized: shLacZ, 94.43±1.5%; shCDKL5#1, 61.13±3.3%; shCDKL5#2, 73.1±5.52%; S3 Fig). Interestingly, at DIV7 the defect could mostly be ascribed to neurons with a surplus of axons (shLacZ: 3.25±1.25%, shCDKL5#1: 38.35±2.88%; shCDKL5#2: 20.4±9.4%) whereas the number of neurons with no axon was decreased (shLacZ: 1.73±1.02%; shCDKL5#1: 4.30±1.48%; shCDKL5#2: 6.5±2.65%) suggesting that the underlying molecular defects are distinct and differently influenced by neuronal maturation.

**Fig 4:**
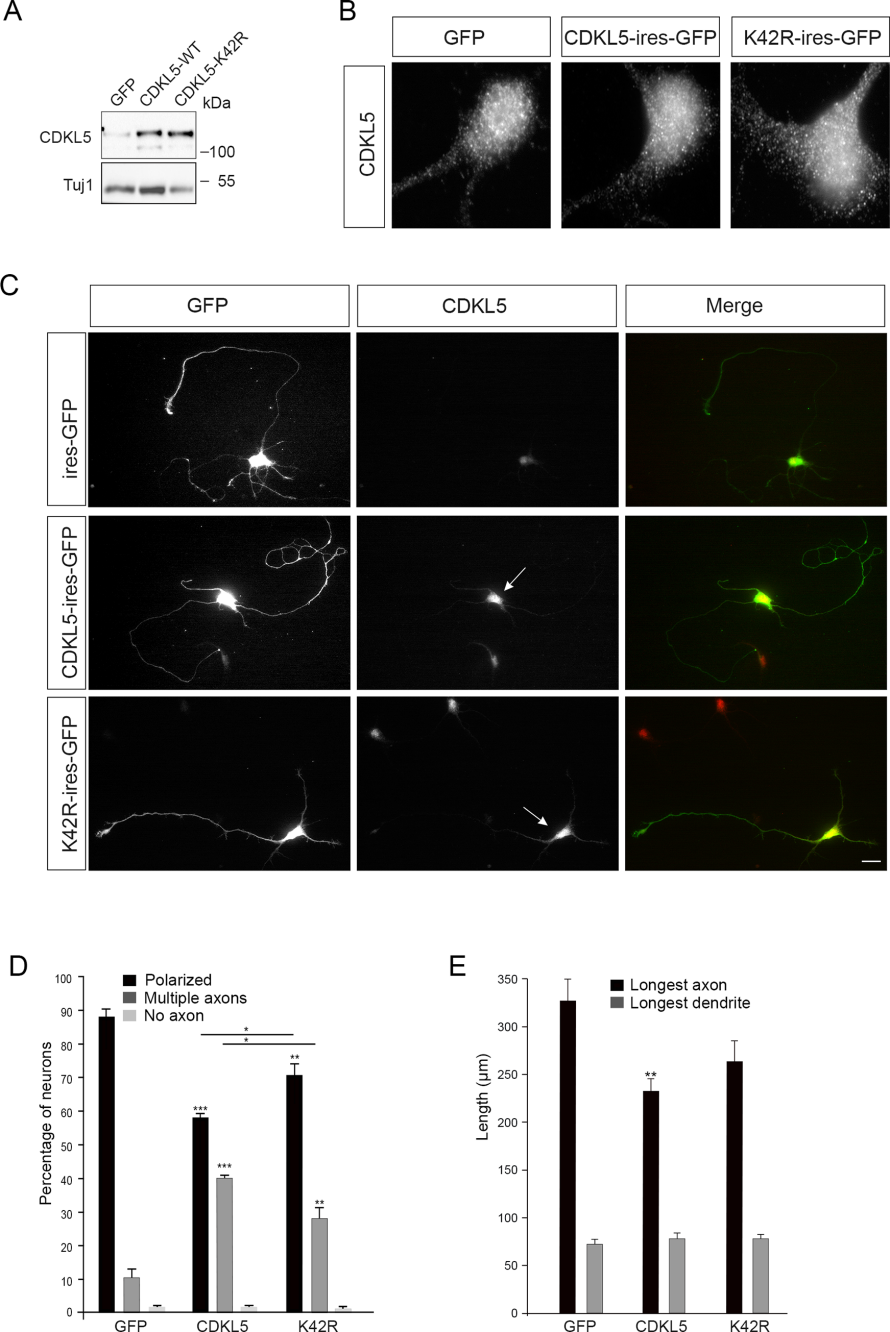
CDKL5 promotes axon formation. (A) Western blot showing CDKL5 levels in primary hippocampal neurons nucleofected before plating with bicistronic vectors expressing GFP alone or together with CDKL5 or CDKL5-K42R. Cell lysates were prepared at DIV5 and analyzed for CDKL5 levels using Tuj1 as loading control. (B) Representative images showing the localization of endogenous and exogenous CDKL5 in the soma of nucleofected GFP-positive neurons at DIV5. The exposure time of the GFP-expressing neuron (left) was increased to reveal the staining of endogenous CDKL5. (C) Representative images showing hippocampal neurons at DIV5 transfected with vectors expressing GFP together with CDKL5 or the K42R derivative. GFP and CDKL5 signals are in green and red, respectively. The arrows indicate neurons with increased CDKL5 levels. Scale bar: 20 μm. (D) Quantitative analysis of neuronal polarization. Axon specification was analyzed at DIV5 by determining the number of neurons with a single axon (polarized, black bars), multiple axons (dark grey bars) and neurons with no axon (light grey bars). Data are expressed as mean of 4 independent experiments ±SEM; ***p<0,001, **p<0,01, *p<0,05 (n≥100 neurons/condition, Student’s *t* test). (E) Graph showing the length of the longest axon and dendrite of transfected neurons. Data present neurite length as means ±SEM (n>28 neurons/condition, 4 independent experiments); **p<0,01. (Student’s *t* test).

We next analyzed the effect of increased CDKL5 levels on neuronal polarization. To investigate the relevance of the catalytic activity of CDKL5 we also expressed a kinase-dead derivative (K42R), carrying a mutation in a critical residue of the ATP-binding site [4]. Primary hippocampal neurons were thus nucleofected at the time of plating with a construct expressing mCDKL5 (or the kinase-dead mutant) and GFP from a bicistronic cassette. The GFP-vector without CDKL5 was used as control. CDKL5 expression and neuronal morphology were analyzed at DIV5 by western blotting and immunofluorescence (IF; Fig 4A–4C). By IF we verified that GFP-positive transfected cells expressed increased amounts of CDKL5 and that the exogenously expressed CDKL5-derivatives displayed the same typical dot-like staining in the soma of the endogenous protein (Fig 4B and 4C). Most of the GFP-positive control neurons were correctly polarized extending only a single axon (means ±SEM: 88.15±2.3%) with an average length of 326±25.8 μm (Fig 4C–4E). On the contrary, the morphology of GFP-positive CDKL5 overexpressing neurons was significantly altered as we detected a large number of neurons, harboring several long Tau1-positive processes (means ±SEM: polarized, 58.36±1.21%; multiple axons, 40.56±0.3%). The mean length of the longest axon (232±14,97 μm) was significantly shorter than that of control neurons (Fig 4E). Multiple axons were also detected in neurons expressing the kinase dead CDKL5-K42R derivative (means ±SEM: polarized, 70.88±3.33%; multiple axons, 28.38±3.2%), but the effect was significantly reduced when compared to neurons expressing the wild-type protein (Fig 4D). Altogether, these data show that CDKL5 promotes axon formation and that its catalytic activity is involved.

### CDKL5 and shootin1 work in common pathway

**Fig 5:**
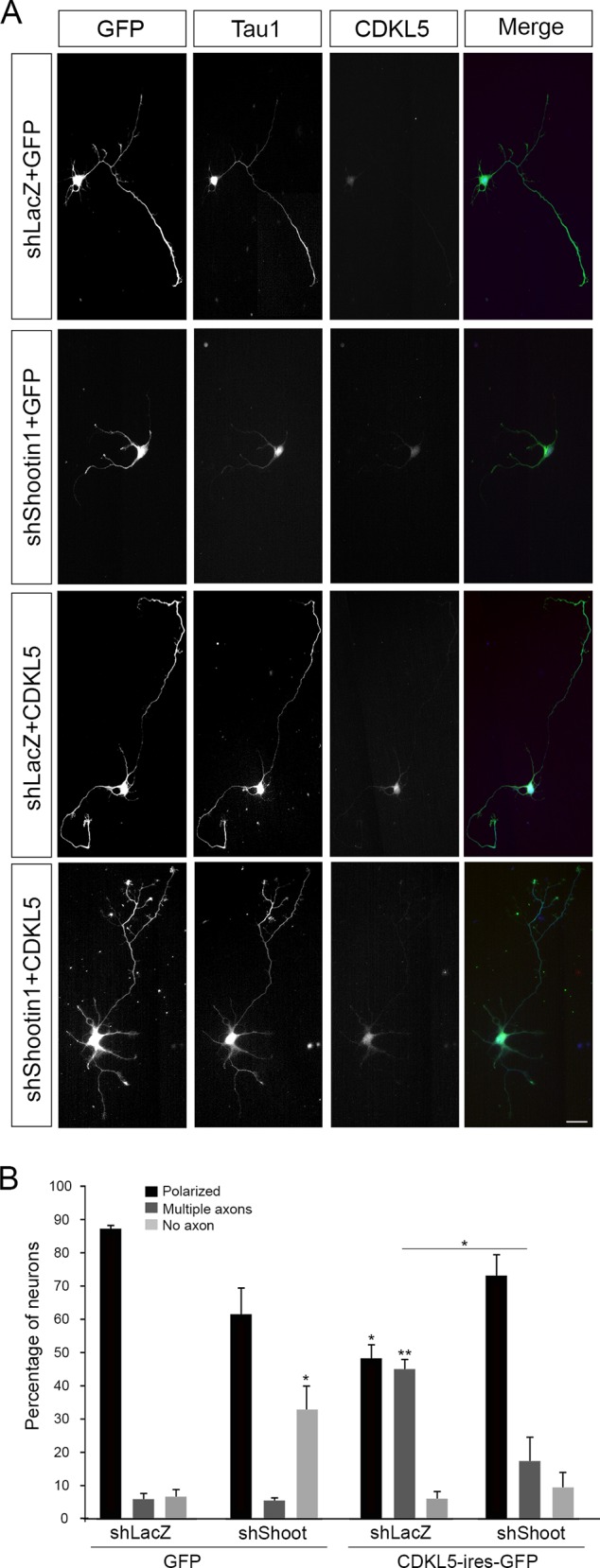
CDKL5 regulates axon outgrowth through shootin1. (A) Immunofluorescence of neurons nucleofected before plating with a bicistronic vector expressing GFP alone or together with CDKL5 and subsequently infected with lentiviral particles expressing shRNAs against shootin1 or LacZ. At DIV5 neurons were stained for GFP, Tau1, and CDKL5 (green, blue, and red, respectively). (B) Quantification of neuronal polarization of GFP-positive neurons with increased CDKL5 expression. Data are expressed as means ±SEM. **p<0,01, *p<0,05. (n≥24 neurons/condition in 3 independent experiments; ANOVA two-way). Scale bar: 20 μm.

The above data suggest that CDKL5 might, at least partially, regulate axon formation through its association with shootin1. Therefore we asked whether the two proteins work in a common molecular pathway. We hypothesized that CDKL5 might promote axon specification only in the presence of shootin1, thus working upstream of shootin1. We therefore overexpressed CDKL5 in neurons in which shootin1 was silenced and analyzed neuronal polarization at DIV5. Neurons were nucleofected at the day of plating with CDKL5- or GFP-expressing vectors and, after another ten hours, infected with lentiviral particles expressing shShootin1#1 or shLacZ as control. As expected, a significant number of neurons expressing shLacZ and exogenous CDKL5 extended more than one axon (Fig 5A and 5B; means ±SEM: polarized, 48.5±4.11; multiple axons, 45.37±2.93), but this effect was significantly attenuated by the silencing of shootin1. Indeed, depletion of shootin1 in conjunction with exogenous CDKL5 expression significantly reduced the number of neurons with a surplus of axons (means ±SEM: polarized, 73.2±6.18; multiple axons 17.37±6.98). As control, we confirmed that the silencing of shootin1 in GFP-expressing neurons generated an increased number of neurons with no axon (means ±SEM: polarized, 61.5±8.09; no axon, 32.67±7.52).

These experiments demonstrate that CDKL5 induces multiple axons only when proper levels of shootin1 are present, suggesting that indeed CDKL5 works upstream shootin1 in the regulation of axon specification.

**Fig 6:**
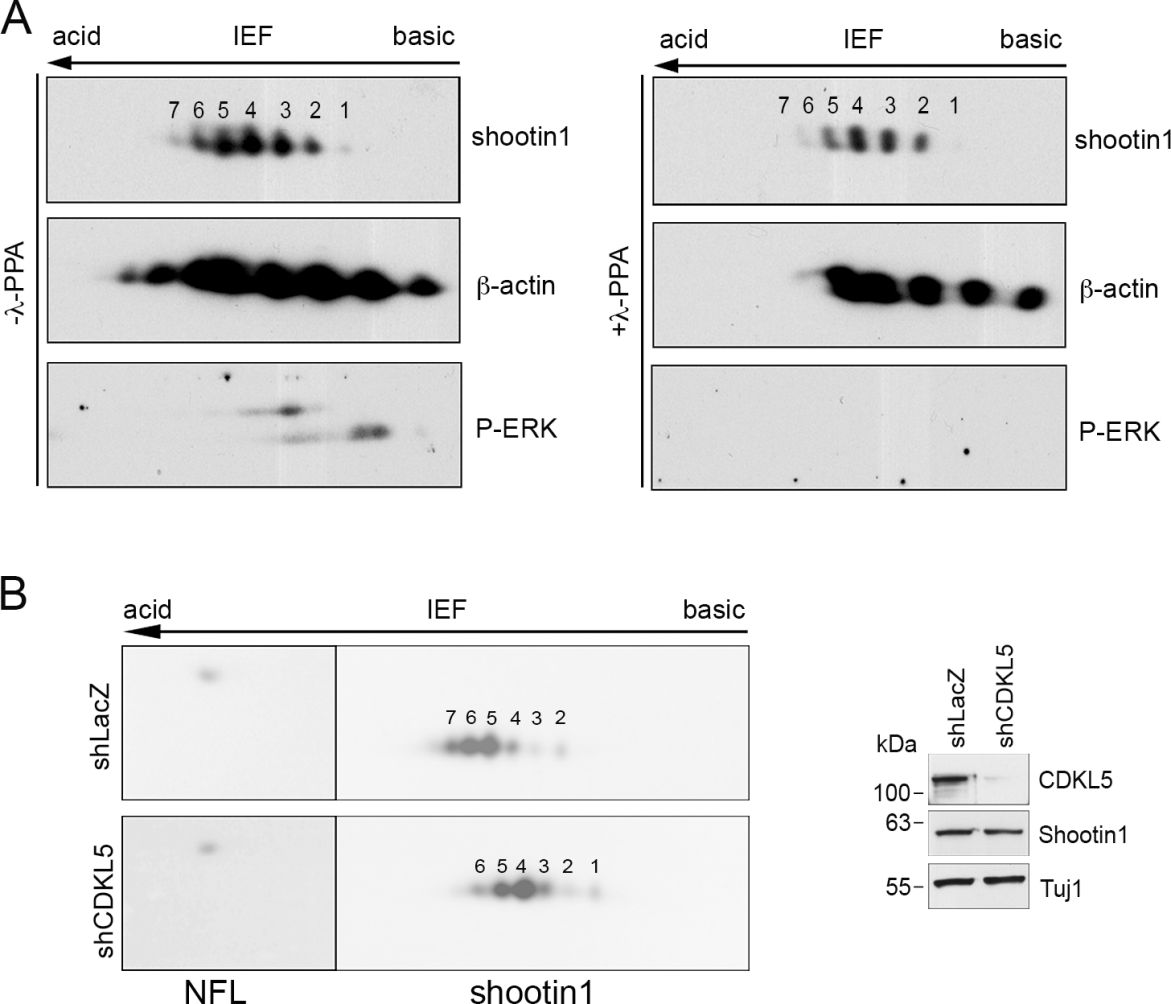
CDKL5 influences shootin1 phosphorylation in primary cortical neurons. (A) Total cell extracts of DIV7 cortical neurons were treated with or without lambda phosphatase (λ-PPase) and analyzed by two-dimensional gel electrophoresis and immunoblotting with antibodies against shootin1, β-actin and, as control for the λ-PPase treatment, phopho-ERK1/2. (B) Primary cortical neurons were infected with shLacZ- or shCDKL5#1-expressing viral particles at DIV0 and total cell lysates were prepared at DIV7 and subjected to two-dimensional gel electrophoresis. Shootin1 and NFL were detected by immunoblotting; the single NFL-spot was used as internal control for alignment. Silencing of CDKL5 was confirmed by western blot (right panel). (n = 3).

Previous reports stated that shootin1 is phosphorylated in hippocampal neurons [23] and, recently, that its PAK1-mediated phosphorylation regulates the traction forces required for axonal outgrowth [24]. We hypothesized that CDKL5 regulates shootin1 activity in a phosphorylation-dependent manner. Therefore, we first analyzed whether the phosphorylation of shootin1 in primary cortical neurons could be detected by two-dimensional SDS-polyacrylamide gel electrophoresis (SDS-PAGE). As shown in Fig 6A (upper panel), shootin1 was resolved in seven distinct spots with different isolectric points. Importantly, samples treated with lambda phosphatase (λ-PPase; lower panel) showed reduced intensity of the two most acidic spots (6 and 7), confirming that they correspond to phospho-isoforms of shootin1.

We then silenced the expression of the kinase by infecting cortical neurons at the time of plating with shCDKL5#1-expressing lentiviral particles to analyze the role of CDKL5 in the post-translational modifications of shootin1 using the single spot of neurofilament (NFL) as internal standard for alignment. Interestingly, a reduction in signal intensity of the two most acidic spots, corresponding to the two shootin1 phospho-isoforms, was easily detected when CDKL5 was silenced (Fig 6B). Consistently, the phosphorylation profile of shootin1 generated from cortices of *Cdkl5*-null mice [13] was altered compared to that of control animals, with a clear accumulation of the more basic isoforms when the kinase was missing (S4 Fig).

Altogether, these experiments confirm a model according to which CDKL5 works upstream shootin1, directly or indirectly affecting its phosphorylation.

## Discussion

Mutations in the X-linked *CDKL5* gene have been identified in patients with the early onset seizure variant of Rett syndrome and other neurological disorders sharing common features such as the appearance of intractable seizures during the first months of life, infantile spasms, hypotonia, and severe intellectual disabilities [2]. The biological functions of CDKL5 are barely understood and only recently the neurological dysfunctions underlying the etiology of CDKL5 associated disorders have become clearer [1]. However, the expression pattern of CDKL5 suggests that it plays a role during early steps of post-natal differentiation and this might, at least in part, fit with the precocious symptoms of CDKL5-related pathologies. Neuronal development relies on a finely tuned interplay between polarization and migration leading to the acquirement of the final neuronal specificity, independently of the neuronal type or brain area analyzed. Subtle deficits in such processes are associated with both epilepsy and autism. It is thus intriguing to speculate that both these features, which represent characteristic phenotypes of patients with mutations in *CDKL5*, might, to some extent, originate from such deficits. A delay in the migration of cortical neuronal precursors devoid of CDKL5 has already been reported [9,10]. This supports the idea that defective CDKL5 activity might affect early steps of differentiation by altering molecular processes resulting in a delayed refinement of cortical architecture, besides impacting on cytoskeletal modifications. Moreover, recent observations reporting that CDKL5 regulates neuronal morphology in cultured neurons [9,10] have been further extended in vivo by the observation that cortical and hippocampal neurons in *Cdkl5*-null brains display reduced arborization [13,25].

Here we show that proper CDKL5 levels are crucial also for axonal specification and elongation. The silencing of CDKL5 results in an increased number of neurons bearing no axon while both silencing and overexpression of the kinase are associated with the formation of supernumerary axons and reduced axonal length. This is the first time that a role of CDKL5 in neuronal polarization is reported. Nevertheless, it is relevant to mention that a role in regulating neurite outgrowth, rather than axon specification, has previously been reported for CDKL5 [9]. We believe that the different timing of the silencing/overexpression and morphological analyses and the variability between cortical rat and hippocampal murine neuronal cultures [26] may explain the different results. In agreement with our data, showing that increased CDKL5 levels induce the formation of a surplus of axons, duplications of genomic regions encompassing *CDKL5* have recently been identified in patients with neurological deficits that include autistic traits [27].

Since the molecular mechanisms by which CDKL5 regulates neuronal functions are far from understood, we aimed at identifying novel CDKL5 interactors that might help addressing this point. In a yeast two-hybrid screening we identified shootin1 as a novel interactor of CDKL5. Shootin1 is a brain-specific protein acting as a determinant of axon formation during the process of neuronal polarization. In unpolarized rat hippocampal neurons, shootin1 randomly fluctuates in the growth cones of multiple neuritis, eventually accumulating in a single neurite before its outgrowth as an axon [17]. Importantly, silencing and overexpression studies have established that shootin1 accumulation in the axon-to-be is both necessary and sufficient for axon outgrowth. By analyzing in details the functional interplay between shootin1 and CDKL5, we found evidence that CDKL5 is involved in regulating neuronal polarization, at least in part, through its interaction with shootin1. We observed low but detectable levels of CDKL5 in unpolarized hippocampal neurons and a strong induction in the transition from stage 2 to stage 3, where it is present in the distal tip of neurites or outgrowing axons together with shootin1. Moreover, we find that the two proteins associate in vivo in early post-natal brains and that correct CDKL5 levels are required for proper axon formation. Similarly to shootin1, the majority of hippocampal neurons over-expressing CDKL5 generates a surplus of axons, whereas a significant number of neurons devoid of the kinase fails to correctly differentiate a single axon. Importantly, the polarization defects observed in neurons with altered CDKL5 expression can be ascribed, at least partially, to a functional interplay with shootin1. In fact, the generation of supernumerary axons induced by CDKL5 is significantly attenuated when shootin1 levels are reduced, confirming that the two proteins act in a common pathway and that CDKL5 acts upstream shootin1. Consequently, we speculated that CDKL5 might influence shootin1 phosphorylation. This post-translational modification of shootin1 is known to be induced by the activation of both Pak1 and cdc42/Rac1 and is required for its association with the actin retrograde flow, allowing the formation of traction forces at axonal growth cones [24]. To support this hypothesis, the catalytically inactive form of CDKL5 (K42R) causes an attenuation of the polarization phenotype induced by the wild-type kinase, suggesting that the catalytic activity of CDKL5 is, at least in part, involved in the observed polarization defects. Moreover, shootin1 phosphorylation is reduced both in neurons silenced for CDKL5 as well as in *Cdkl5*-null cortices, reinforcing the idea that the two proteins work in a common molecular pathway.

As a possible scenario, we hypothesize that the absence of CDKL5 reduces the traction force by altering the phosphorylation state of shootin1, thus causing a reduction of axon length. Whether CDKL5 phosphorylates shootin1 directly or through an intermediary kinase remains to be revealed as well as the specific residues of shootin1 whose modification depends on CDKL5. It is interesting to note that Rac1 appears to be common to the pathways of both CDKL5 and shootin1. As mentioned, shootin1 phosphorylation is induced by cdc42/Rac1-dependent Pak1 activation and CDKL5 has been reported to regulate neuronal morphology acting upstream Rac1 [9,24]. In the future, it will be interesting to analyze whether CDKL5 exerts its regulatory functions on shootin1 in a Rac1-dependent manner.

Even if the main body of our experiments is in line with a role of CDKL5 in neuronal polarization through its association with shootin1, the picture is complicated by the fact that silencing of CDKL5 generates a mixed phenotype including both neurons devoid of axons and neurons with numerous axons. CDKL5 is very likely to influence different downstream pathways through its different interaction partners and may thus target different factors involved in regulating axon specification and outgrowth. In this scenario, we believe that CDKL5 influences neuronal polarization in part through shootin1 and in part in a shootin1-independent manner. Disruption of the shootin1-dependent mechanism generates neurons devoid of axons and a concomitant reduction in axon elongation of properly polarized neurons. This defect seems to be a mere delay in axon outgrowth and elongation since the percentage of neurons with no axon returns to control levels at DIV7, as also reported for neurons with reduced shootin1 levels [24]. Conversely, the impairment of the other pathway, which still remains to be identified, results in excessive axon formation and persists at DIV7.

A kinome profiling of brain extracts from *Cdkl5*-null mice revealed that several signaling pathways are deregulated in its absence [12]. Interestingly, the three pathways that were mostly influenced were those including AMPK kinase, PKA, and AKT that are all involved in neuronal polarization [12]. Accordingly, the activity of GSK3-β, a downstream effector of AKT, is altered in *Cdkl5*-null mice [25], whereas, a pleiotropic effect of GSK3-β on neuronal polarization has already been demonstrated [28]. In the future, it will be intriguing to elucidate the contribution of GSK3β in the polarization defects associated with CDKL5. Moreover, as mentioned, CDKL5 works upstream of Rac1 [9] and it is well established that Rac1 influences various aspects of neuronal morphogenesis through different upstream regulators and downstream effectors. Axon initiation is mediated by Rac1 in a pathway involving DOCK7 and stathmin whereas this small Rho GTPase appears to have a dual role in regulating axon outgrowth. Its inhibitory activity requires a pathway including PAK, LIMK, and cofilin whereas growth promotion is PAK-independent [29]. Finally, it is possible that the silencing of CDKL5 has different efficiency and/or timing in different neurons thus influencing its downstream effectors in distinct time windows with opposing outcomes.

Altogether, our data indicate a so far uncharacterized role of CDKL5 in the early phases of neuronal differentiation that may concur to the clinical features associated with mutations in this gene. Finally, both the association of shootin1 with a molecular pathway belonging to CDKL5 and the defects in neuronal migration previously associated with its deficiency [30] might make shootin1 an interesting candidate gene for neurological disorders.

## Supporting Information

S1 Fig CDKL5 localization in neurites and axons of hippocampal neurons. (A) Immunofluorescence analysis of primary hippocampal neurons expressing GFP (green) at stages 2–3 with anti-CDKL5 (red). Quantitative profiles showing the fluorescence intensities of GFP (green) and CDKL5 (red) from the soma to the distal tip of the neurites/axons indicated with asterisks are shown to the right. CDKL5 is present in the distal tip of the axon without showing a specific accumulation. (B) Hippocampal neurons were infected at the day of plating with lentiviral particles expressing two different shRNAs against CDKL5 or, as control, against LacZ. At DIV4, neurons were stained for CDKL5 (red); GFP is in green. The signal intensity of CDKL5 was analyzed with ImageJ and the mean values plotted in the graph to the right. n = 30.
(TIF)

S2 Fig Aberrant neuronal polarization in the absence of CDKL5. Immuofluorescence of DIV4 hippocampal neurons expressing shRNAs against LacZ or CDKL5. Polarized neurons extend one Tau1-positive and MAP2-negative axon. Neurons silenced for CDKL5 present supernumerary axons (middle column) or do not extend any axon (right column). Tau1, MAP2 and GFP are in blue, red and green, respectively.
(TIF)

S3 Fig Neuronal polarization defects caused by CDKL5 silencing persist at DIV7. Graphs showing polarization of hippocampal neurons expressing shRNAs against CDKL5 or LacZ at DIV4 and DIV7. The graphs show the percentage of polarized neurons, neurons with multiple axons and with no axon at the two different time points as means ±SEM (n≥3, a total of >100 neurons were analyzed). ***p<0,001, **p<0,01, *p<0,05. (Student’s *t* test).
(TIF)

S4 Fig Altered shootin1 phosphorylation in *Cdkl5*-null cortices. Cortices of P5 wild-type and *Cdkl5*-null mice were lysed in Tris pH 7.4, NaCl 150 mM, CHAPs 0.5%, EDTA 0.2 mM, PMSF 1 mM, inhibitors of proteases and phosphatases and treated or not with λ-PPase. Nucleic acids and lipids, possibly interfering with 2-DE, were removed by centrifugation in ultrafiltration concentration devices (Vivaspin 500 MWCO 3000 Da PES, Sartorius) at 15000 g to a final volume of 100 μl. Samples were subsequently diluted in UTC (7 M urea, 2 M thiourea, 4% CHAPS) and 200 μg of proteins were subjected to isoelectric focusing. The subsequent immunoblotting was performed first against shootin1 and next against neurofilament (NFL; MA5-14981, Thermo Scientific, diluted 1:1000 in 5% non-fat milk in TBST) without stripping the membrane for the anti-shootin1 antibodies. The NFL signal could thus be used as internal standard for alignment. n = 2.
(TIF)
