# Supplementary figures and images for: CDKL5 and Shootin1 Interact and Concur in Regulating Neuronal Polarization
Source: PLoS One. 2016 Feb 5;11(2):e0148634. doi: 10.1371/journal.pone.0148634 (PMC4746202; doi:10.1371/journal.pone.0148634)

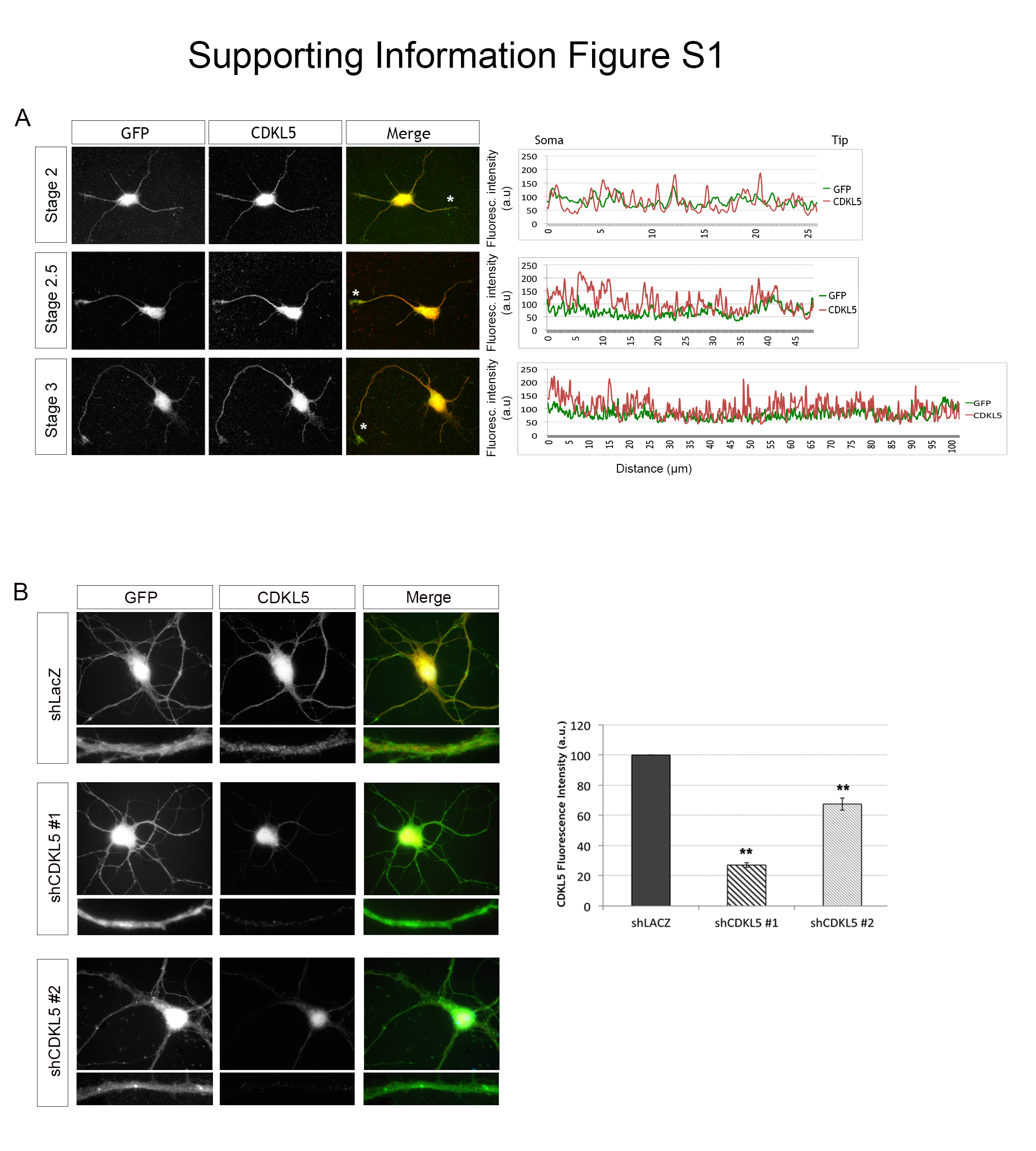

Supplement: S1 Fig — (A) Immunofluorescence analysis of primary hippocampal neurons expressing GFP (green) at stages 2–3 with anti-CDKL5 (red). Quantitative profiles showing the fluorescence intensities of GFP (green) and CDKL5 (red) from the soma to the distal tip of the neurites/axons indicated with asterisks are shown to the right. CDKL5 is present in the distal tip of the axon without showing a specific accumulation. (B) Hippocampal neurons were infected at the day of plating with lentiviral particles expressing two different shRNAs against CDKL5 or, as control, against LacZ. At DIV4, neurons were stained for CDKL5 (red); GFP is in green. The signal intensity of CDKL5 was analyzed with ImageJ and the mean values plotted in the graph to the right. n = 30. (TIF) [file pone.0148634.s001.tif]

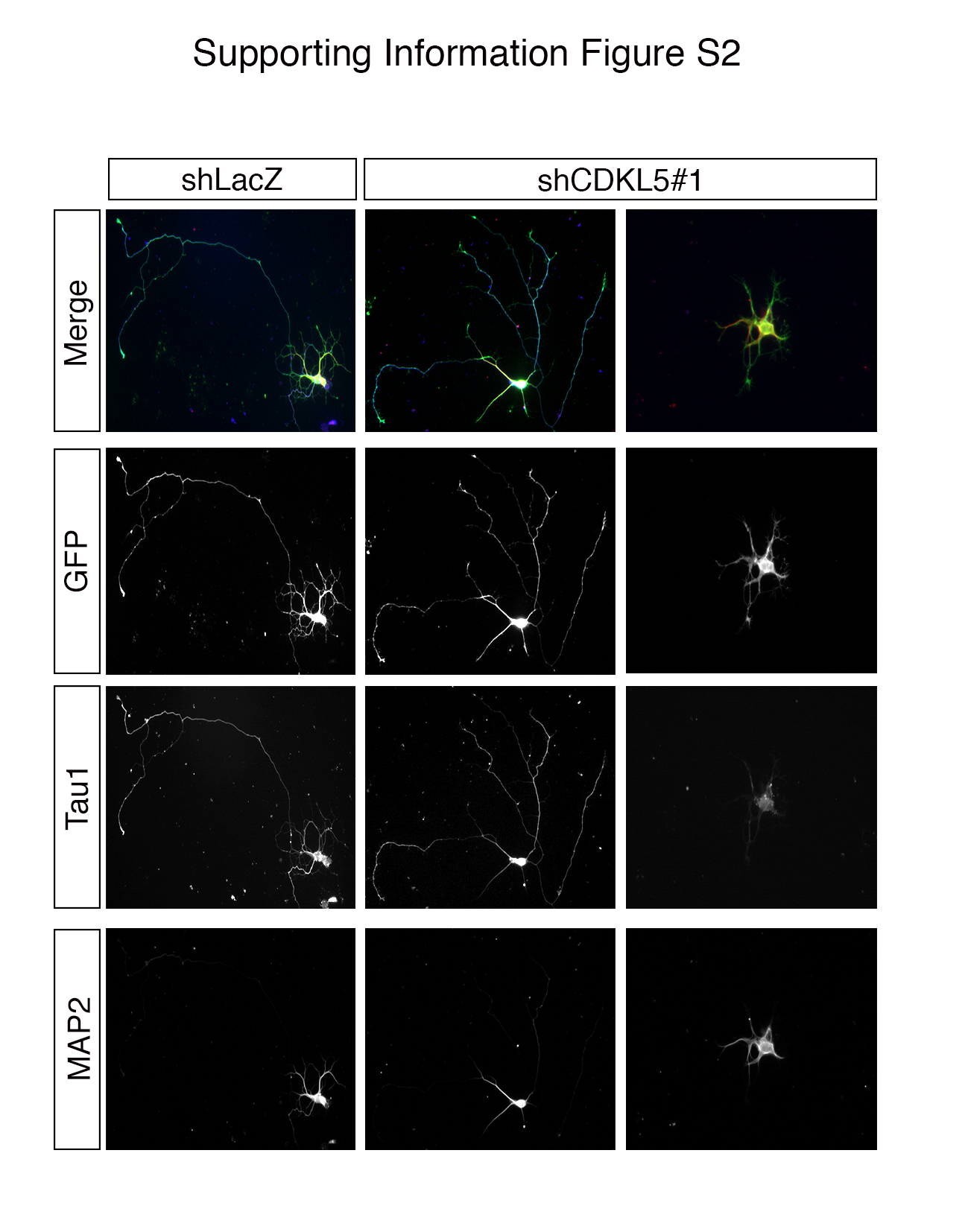

Supplement: S2 Fig — Immuofluorescence of DIV4 hippocampal neurons expressing shRNAs against LacZ or CDKL5. Polarized neurons extend one Tau1-positive and MAP2-negative axon. Neurons silenced for CDKL5 present supernumerary axons (middle column) or do not extend any axon (right column). Tau1, MAP2 and GFP are in blue, red and green, respectively. (TIF) [file pone.0148634.s002.tif]

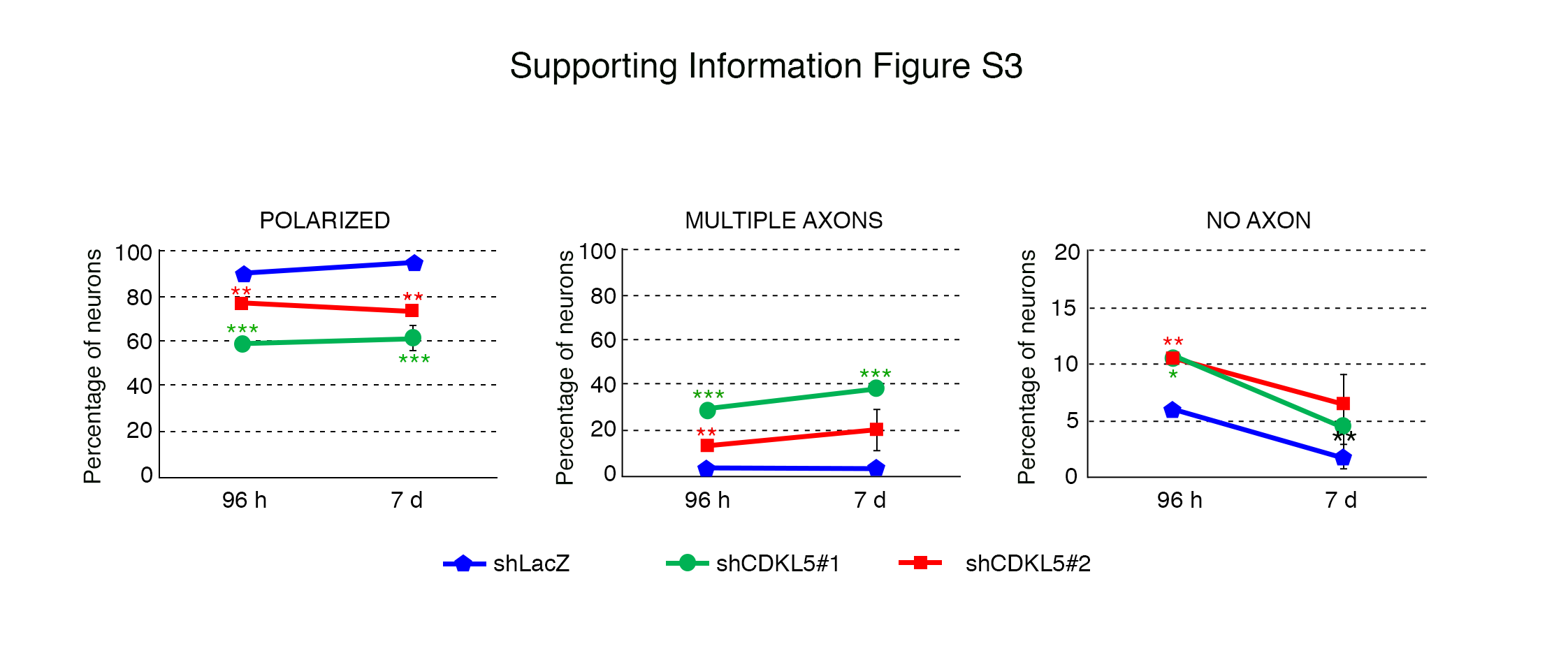

Supplement: S3 Fig — Graphs showing polarization of hippocampal neurons expressing shRNAs against CDKL5 or LacZ at DIV4 and DIV7. The graphs show the percentage of polarized neurons, neurons with multiple axons and with no axon at the two different time points as means ±SEM (n≥3, a total of >100 neurons were analyzed). ***p<0,001, **p<0,01, *p<0,05. (Student’s t test). (TIF) [file pone.0148634.s003.tif]

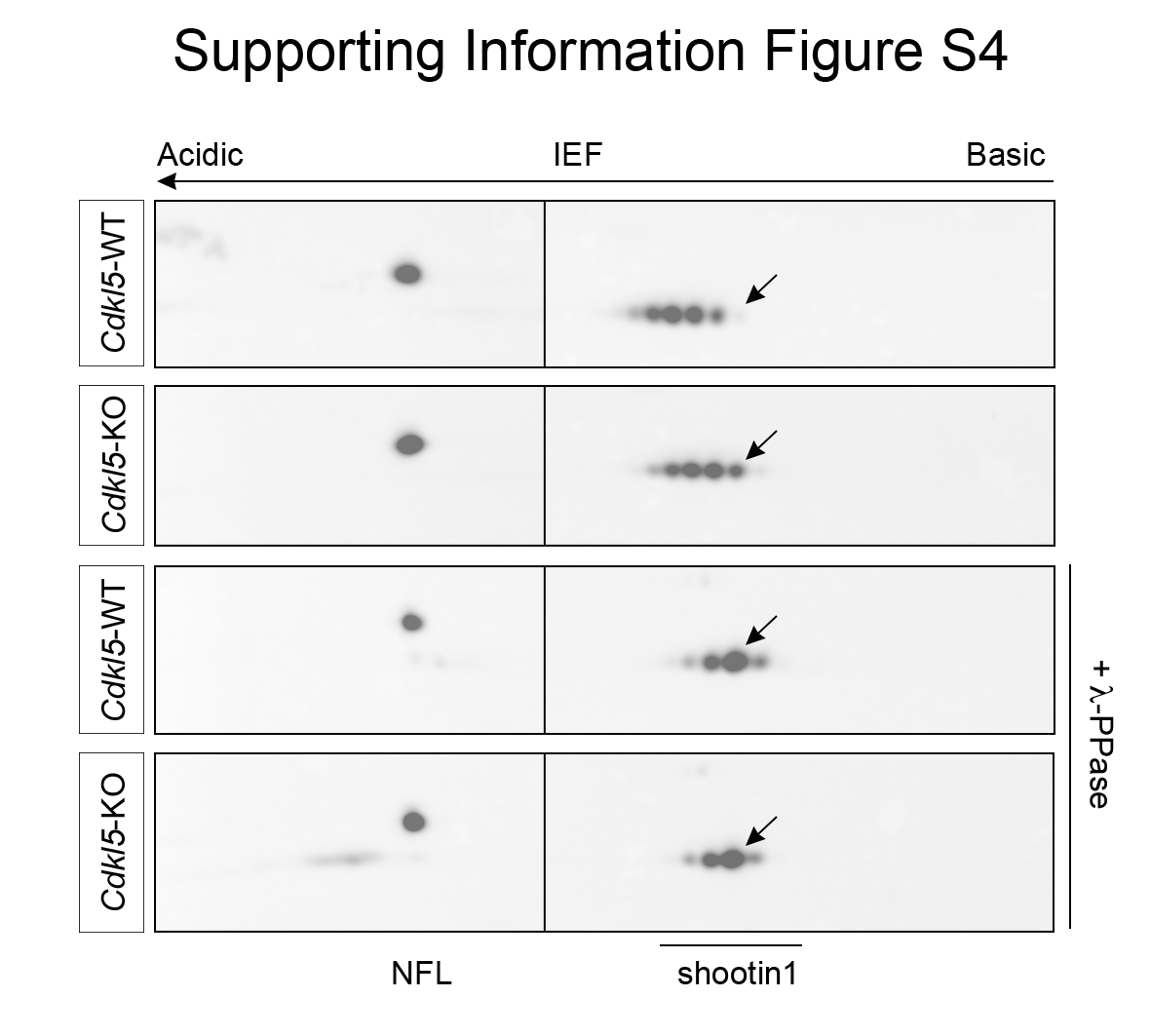

Supplement: S4 Fig — Cortices of P5 wild-type and Cdkl5-null mice were lysed in Tris pH 7.4, NaCl 150 mM, CHAPs 0.5%, EDTA 0.2 mM, PMSF 1 mM, inhibitors of proteases and phosphatases and treated or not with λ-PPase. Nucleic acids and lipids, possibly interfering with 2-DE, were removed by centrifugation in ultrafiltration concentration devices (Vivaspin 500 MWCO 3000 Da PES, Sartorius) at 15000 g to a final volume of 100 μl. Samples were subsequently diluted in UTC (7 M urea, 2 M thiourea, 4% CHAPS) and 200 μg of proteins were subjected to isoelectric focusing. The subsequent immunoblotting was performed first against shootin1 and next against neurofilament (NFL; MA5-14981, Thermo Scientific, diluted 1:1000 in 5% non-fat milk in TBST) without stripping the membrane for the anti-shootin1 antibodies. The NFL signal could thus be used as internal standard for alignment. n = 2. (TIF) [file pone.0148634.s004.tif]
